# Defying barriers to fight tuberculosis in West Africa: a model of equitable partnerships within a research capacity-strengthening network in the subregion

**DOI:** 10.3389/fpubh.2025.1590282

**Published:** 2025-09-19

**Authors:** Isaac Darko Otchere, Victory Fabian Edem, Toyin Togun, Dorothy Yeboah-Manu, Martin Antonio, the WANETAM-TB-Network

**Affiliations:** ^1^Noguchi Memorial Institute for Medical Research, University of Ghana, Accra, Ghana; ^2^Medical Research Council Unit The Gambia at London School of Hygiene and Tropical Medicine, Banjul, Gambia; ^3^Department of Clinical Research, Faculty of Infectious and Tropical Diseases, London School of Hygiene and Tropical Medicine, London, United Kingdom; ^4^The TB Centre, London School of Hygiene and Tropical Medicine, London, United Kingdom; ^5^Department of Infection Biology, Faculty of Infectious and Tropical Diseases, London School of Hygiene and Tropical Medicine, London, United Kingdom; ^6^Centre for Epidemic Preparedness and Response, London School of Hygiene and Tropical Medicine, London, United Kingdom

**Keywords:** Tuberculosis, West Africa, WANETAM, Capacity building, end TB

## Abstract

Tuberculosis (TB), caused by the *Mycobacterium tuberculosis* complex (MTBC), is the leading cause of death from a single infectious disease, despite being treatable. Global TB control efforts face significant challenges, including insufficient funding, ineffective vaccines, inadequate diagnostics, and complex treatments, particularly in resource-limited regions. West Africa has a unique TB epidemiology, characterized by medium- to high-prevalence rates and a greater diversity of the MTBC, which further compounds control efforts. In response to the global call to end TB by 2030, the West African Network of Excellence for TB, AIDS, and Malaria (WANETAM) has united scientists from 25 institutions across 12 West African countries to build research capacity and conduct translational research focused on TB. The multi-country program of WANETAM focuses on assisting the TB control programs of its member countries through the supply of essential laboratory equipment and the facilitation of laboratory accreditation/certification (with three regional laboratories already ISO accredited and others on track). The program also emphasizes gender-sensitive training, the retention of critical laboratory and research expertise, improvements in the diagnosis of TB (including pediatric and drug-resistant forms), and conducting studies on TB to better understand the molecular epidemiology of the MTBC in the sub-region, thereby generating the evidence to inform the policy. To date, WANETAM has trained 13 postdoctoral fellows, 753 laboratory technicians/technologists, and currently supports 7 female PhD students in the final year of their respective programs. By fostering collaboration among Francophone, Anglophone, and Lusophone West Africans, WANETAM is bridging cultural and language barriers to fight TB while also preparing West Africa for future pandemics.

## Introduction

Tuberculosis (TB), caused by the *Mycobacterium tuberculosis complex* (MTBC), is an ancient disease that has blighted mankind since the beginning of recorded history (1). Although treatable, TB has been the leading cause of mortality from a single infectious disease for the past decade—relinquishing this position to COVID-19 from 2020 to 2022, before reclaiming it in 2023 (1–4). TB remains a global public health emergency due to several factors, including the limited effectiveness of the only World Health Organization (WHO)-approved Bacillus Calmette Guerin (BCG) vaccine, suboptimal diagnostics, and the emergence of drug-resistant strains of MTBC, which threatens to make TB untreatable. In 2022, the WHO estimated 10.6 million new TB cases (with 7.5 million actual notifications), including 1.3 million children under 15 years, while as many as 1.3 million people died from TB (including 167,000 among people living with HIV/AIDS) (1). An estimated 410,000 cases of TB were caused by MTBC that is resistant to rifampicin, which is the backbone of the Directly Observed Treatment Short-Course (DOTS) regimen (1). Approximately 65% of the global TB burden is attributed to five major risk factors: undernutrition, HIV/AIDS, alcohol use/disorders, smoking, and diabetes mellitus, with undernutrition alone accounting for 30%. With Africa having the highest rate of undernutrition (21.7%), it is therefore not surprising that the continent carries approximately 24% of the global TB burden, despite being home to only 15.19% of the global human population (1, 5, 6).

The epidemiology of TB in West Africa is characterized by a medium- to high-prevalence of TB. Nigeria, Liberia, and Sierra Leone are among the 30 countries with the highest TB burden globally, while Nigeria, Liberia, Guinea, and Guinea-Bissau are also among the 30 countries with a high TB/HIV burden worldwide (1). In addition, the burden of multidrug-resistant (MDR) TB is high in West Africa, with Nigeria listed among the 30 countries with the highest burden for this condition.

The control of TB took a negative turn in 2022, with case detection and treatment enrollment rates declining from the pre-COVID-19 pandemic positive outlook (7). This led to the WHO and the STOP TB Partnership calling for strategically deliberate investments to help “End TB to save lives,” as the global attention remained focused on the COVID-19 pandemic (3). This call reminded the world that TB is still globally relevant, killing millions of people in every WHO region, and its control programs needed all the necessary resources to ensure that the successes and gains achieved over the years would not be reversed. In 2023, the theme for World TB Day was “Yes! We Can End TB,” which was repeated for the year 2024. In 2025, World TB Day carries the same theme “Yes! We Can End TB: Commit, Invest, Deliver,” emphasizing that a world free of TB can only be achieved through deliberately bold and collective efforts. It is a call for continued commitment and increased investment from governments, healthcare providers, and communities worldwide to provide resources, fund research, and deliver better healthcare solutions to combat TB, aiming for its elimination by 2030.

**Figure 1:**
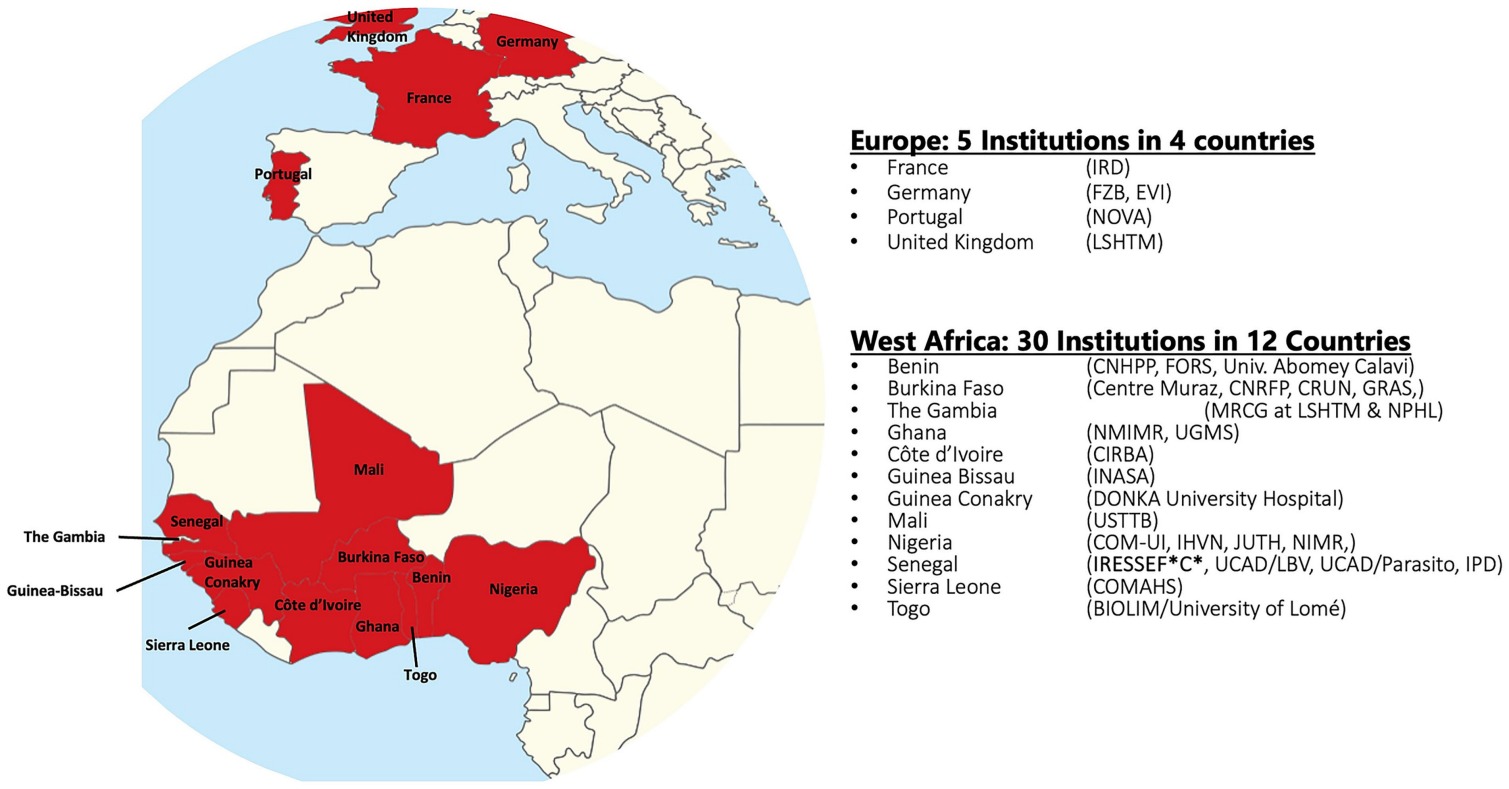
Global map of countries with institutional membership of WANETAM.

In response to the call by the WHO and the STOP TB Partnership to invest in TB eradication, we describe in this study what the West African Networks of Excellence for TB, AIDS, and Malaria (WANETAM), which is funded by the European and Developing Countries Clinical Trials Partnership (EDCTP), has been doing in West Africa to help fight TB while preparing for potential future pandemics.^1^ With three consecutive funding cycles since 2009, WANETAM has bridged language and cultural barriers to bring together leading scientists from 25 institutions in 12 West African countries, including Anglophone, Francophone, and Lusophone countries, in collaboration with 5 institutional partners in 4 European countries (Figure 1). This consortium has been working to upgrade research and laboratory facilities, support laboratory and institutional preparedness for accreditation/certification, train critical expertise required for specialized diagnostics and clinical research, and conduct clinical trials and other public health research to fight today’s problems while preparing for potential future epidemic threats in West Africa.

### Improvement in research administration and infrastructure

When WANETAM was established, we assessed the facilities within the network using the Stepwise Laboratory Improvement Process Toward Accreditation (SLIPTA) framework developed by WHO’s Regional Office for Africa.^2^ This helped us understand the existing strengths and targets for improvement in order to achieve international accreditation. Through targeted interventions and sustained partnerships, WANETAM’s holistic support has elevated partner sites from foundational levels of readiness to states of high proficiency, fostering regional self-reliance in tackling infectious diseases, including TB. Support has been provided across laboratories, clinical trial sites, data systems, and financial frameworks, as shown below (Table 1), positioning these institutions as key players in global health emergency preparedness and research.

**Table 1:** Research and administrative support by WANETAM to West African institutions.

Area of improvement	Name of site	Level at baseline	Current level
Molecular Biology	UCAD HIV	2	WHO-accredited
Biobank	UCAD HIV	1	3
Clinical Trial	UCAD HIV	1	3
Chest Clinic, TB Laboratory	UGMS, Ghana	1	2
TB Reference Laboratory	BHP, Guinea-Bissau	1	3
Laboratories	University of Ibadan, Nigeria	1	3
Reference Laboratory	NIMR, Nigeria	2	4
Clinical trial facilities	UCAD Coordination, Senegal	2	4
	UCAD HIV/TB Senegal	2	4
	University of Ibadan, Nigeria	1	3
	Innovative Biotech, Nigeria	1	3
Data management	UCAD Coordination Senegal	1	4
	UCAD HIV/TB, Senegal	1	4
	University of Ibadan, Nigeria	1	4
Information technology and data sharing facilities	UCAD Coordination, Senegal	2	4
	UCAD HIV/TB Senegal	2	4
	KBTH, Ghana	1	2
	BGP, Guinea-Bissau	1	4
	University of Ibadan, Nigeria	1	4
Finance and administration	UCAD Coordination Senegal	2	4
	UCAD HIV/TB Senegal	2	4
	KBTH, Ghana	1	2
	University of Ibadan, Nigeria	2	4

The grading system used in the table corresponds to the SLIPTA star system, where “1, 2,3, and 4” represent “1 star, 2stars, 3 stars, and 4 stars,” respectively.

A primary focus has been on enhancing foundational laboratory and clinical capabilities. A notable achievement is the elevation of Molecular Biology and TB diagnostic laboratories, along with clinical trial and biobanking facilities at Université Cheikh Anta Diop (UCAD) in Senegal, University of Ghana Medical School (UGMS), University of Ibadan in Nigeria, Nigeria Institute of Medical Research (NMIR), and Bandim in Guinea-Bissau. With the support of WANETAM, these facilities progressed from a developing level to achieving full WHO accreditation or a higher proficiency level. These upgrades have provided the foundation for advanced clinical research and preparedness for future epidemic threats. Beyond the laboratory, WANETAM has also invested in the critical data and IT infrastructure required for modern research at institutions in West Africa. WANETAM has dramatically improved data management systems at the coordination and research sites in Senegal and Nigeria, advancing them from baseline level 1 to expert level 4 to ensure that research data were captured and analyzed according to best practices. This has been complemented by significant improvements in IT and data-sharing facilities at institutions such as the Korle-bu Teaching Hospital (KBTH) in Ghana and the Bandim Health Project (BHP) in Guinea-Bissau, thus creating a more interconnected and collaborative research ecosystem. The institutional support by WANETAM has also extended to essential administrative functions that ensure operational sustainability and good governance. This is highlighted by the support given to UCAD, the University of Ibadan, and the KBTH to enhance their finance and administration services to higher proficiency levels for better grant management and operational integrity.

Through this multifaceted approach, WANETAM has continued to systematically build a resilient and highly skilled network of institutions capable of responding effectively to the region’s public health challenges.

### Training and retention of a gender-sensitive critical mass of expertise in West Africa

Using modeled periodic practical workshops, WANETAM has trained a total of 753 individuals, including 448 men (59.5%) and 305 women (40.5%), intentionally recruited from among the employed staff of public health and academic research institutions across West Africa. Over the 15 years since its establishment, this has been achieved through collaborative research projects and structured training within the thematic Nodes of Excellence (NoEs) for TB, AIDS, malaria, neglected tropical diseases (NTDs), Ebola, and other emerging infectious diseases. Additionally, WANETAM has offered 13 postdoctoral fellowships (ranging between 2 and 4 years) to early-career scientists competitively selected from academic and research institutions in West Africa, to support their transition to independence. To ensure retention of this trained expertise in West Africa, beneficiaries of our training programs are always selected from among individuals with secured employment in West Africa. Additionally, recognizing the gender gap in clinical research capacity and the limited career development opportunities for female researchers in West Africa, we recently initiated a gender-sensitive TALENT postgraduate fellowship program aimed at training female West African scientists at MSc and PhD levels.^3^ This program has already recruited seven female PhD student scientists as the first cohort, following a competitive selection process. To ensure that they can complete their programs without hindrance, the fellowship, in addition to fees and research support, provides accommodation and subsistence support. It further allows for extensions for those who may require additional time due to childbirth or other genuine family responsibilities. The intention is to move these women into our postdoctoral program after they graduate, ensuring continuity within the network and bridging the gender gap. The strategy for training and collaboration within our network is focused on project-based training aimed at building research leadership for hands-on clinical studies, resource and platform infrastructure development for clinical laboratory diagnostics, collaborative research data sharing, conducting evidence-based surveillance needed for designing clinical trials, developing and evaluating diagnostics to support interventions, and building a robust quality assurance management system to support laboratory accreditation/certification.

### Targeted research activities

West Africa has a unique and highly heterogeneous TB epidemiology with varying burdens of TB, as shown in Table 2. The debilitating impact of TB, especially TB-related mortality in West Africa, has declined significantly by 53.1% since 2009, compared with a 32.3% reduction in sub-Saharan Africa and 16.9% globally. However, despite the contributions of WANETAM and other stakeholders, including local governments, regional bodies, and the WHO through the Global Fund since 2009, the TB incidence rate has seen the least reduction in West Africa (17.2%) compared to the entire sub-Saharan African region (38.1%), albeit better than the global reduction of 2.2% (Table 2) over the same period.

**Table 2:** Varying burden of tuberculosis among WANETAM partner countries in West Africa.

Partner country	TB Deaths (thousand)		Estimated TB incidence/100,000/Year		Current population (millions)
	2009	2023	2009	2023	
Benin	1.50	1.20	69	51	13.3
Burkina Faso	8.70	0.71	60	43	22.7
Cote d’Ivoire	18.0	6.30	207	119	28.2
Gambia	0.83	0.47	180	142	2.7
Ghana	11.0	9.90	187	129	33.5
Guinea Bissau	0.49	1.60	361	361	2.1
Guinea Conakry	7.30	2.30	190	175	13.9
Mali	11.0	1.50	64	48	22.6
Nigeria	110	64.0	219	219	218.5
Senegal	9.00	1.80	134	110	17.3
Sierra Leone	9.00	1.30	318	283	8.6
Togo	7.50	0.08	77	30	8.8
Total WANETAM	194.32	91.16	172.2	142.5	392.2
Africa	430	291	333	206	824
Global	1,300	1,080	137	134	6,826

According to the 2010 and 2024 Global tuberculosis reports (4, 29) and the World Bank report (2000–2023).

West Africa is home to six out of the nine known phylogenetic lineages of MTBC, including four lineages (L1, L2, L3, L4) of the generalist *M. tuberculosis sensu stricto* (Mtbss) and two lineages (L5 and L6) of the specialist M*ycobacterium africanum* (Maf), which is geographically restricted to West Africa for reasons that are still unclear (8, 9). Therefore, the WANETAM consortium has been evaluating the WHO-approved diagnostics in West Africa to ensure that such tools are pertinent for TB control in the sub-region. Researchers within the TB work package have spearheaded these studies, including the evaluation of the MTBDR*plus* and MTBDR*sl* line probe assays, respectively, for the detection of TB drug resistance to first-line and second-line drugs (10, 11), and assessing the accuracy of the mpt64-based lateral flow assay for the diagnosis of TB (12, 13). Other studies include the evaluation of the Human TB LAMP diagnostic tool (14) and investigation of the pragmatic diagnostic accuracy of GeneXpert Ultra for the diagnosis of childhood TB in West Africa (15). These studies showed that the MTBDRplus/sl and Human TB LAMP assays were highly sensitive and specific for detecting drug-resistant TB (DR-TB) and diagnosing TB, respectively. On the other hand, the 55% sensitivity of GeneXpert Ultra for diagnosing TB among children under 15 years was significantly below the 95% sensitivity recorded for diagnosing TB among adults (16). Similarly, the mpt64-based lateral flow assay failed to detect 24% of TB caused by L5 compared to 2% of TB caused by L4 MTBC bacteria (13) and is 2.5-fold less likely to detect TB caused by L6 compared to L4 (12). These findings underscore the need to promote TB research into new TB diagnostics and therapeutics in West Africa, where MTBC L5 and L6 are restricted and cause up to 50% of TB in some countries (8).

Given the paucity of research and limited notification data on DR-TB in West Africa, primarily due to the difficulty in diagnosis (17), the WANETAM TB network proactively began training researchers, clinical laboratory, and national TB program (NTP) staff drawn from across the region during the first funding cycle of the consortium between 2009 and 2012 for surveillance. We then carried out a pilot drug-resistant (DR) TB surveillance study in eight West African countries in collaboration with various national TB control programs, which revealed that the burden of MDR-TB within the sub-region may have been underestimated over the years by the WHO (18). While the WHO estimated the prevalence of MDR-TB in new and retreatment cases in the sub-region to be 2 and 17%, respectively, in 2014 (19), our surveillance, which analyzed 974 bacterial isolates from the sub-region, found the prevalence of MDR-TB in new and retreatment cases to be 6 and 35%, respectively (18). Of utmost concern was the observed emergence of pre-extremely-DR (pre-XDR) TB cases that were found in all eight participating countries (18). In addition, during the second cycle of WANETAM, the WANETAM TB network in Ghana, working with the Ghana national TB control program, analyzed sputum collected from difficult-to-treat TB patients (including treatment failures, non-converting follow-up patients, relapsed TB patients, retreatment cases, and known DR-TB patients) from eight regions of the country from 2017 to 2019 (20). The previously mentioned study found 42% cases to be MDR/RR-TB among the 298 confirmed TB cases, including 19 pre-XDR-TB cases (MDR/RR-TB with additional resistance to at least one fluoroquinolone drug). This is significantly higher than the WHO estimates for Ghana over the same period, which have consistently been below 5% among retreatment cases in the country (2, 21, 22).

Ten years have passed since the DR-TB survey by WANETAM in West Africa. Has the actual burden of DR-TB in the sub-region, especially MDR and pre-XDR, changed over the years, even with the re-definition of DR-TB by WHO in 2020? (23). Current WHO estimates show varying burdens of MDR/rifampicin-resistant (RR) TB across West Africa, ranging from 0.50% among new TB cases in Togo to 44% among previously treated TB cases in Guinea-Bissau (4). Based on data from previous studies, it is very likely that the recent WHO estimates do not necessarily reflect the actual burden. Therefore, the WANETAM TB work package is currently conducting an expanded, prospective DR-TB survey at 14 WANETAM TB network partner sites across West Africa to determine the current burden of DR-TB. Second, the current study uses next-generation sequencing (NGS) of the resulting MTBC isolates to determine the genetic drivers of the emergence and potential transmission of DR-TB in the sub-region. Using NGS for studying the evolution of DR among the MTBC is essential because it provides high-resolution whole-genome data, allowing for the detection of low-frequency resistance mutations (heteroresistance) within a single patient, which is critical for understanding the initial emergence and subsequent fixation of resistance (24–27). Furthermore, by comparing single-nucleotide polymorphisms (SNPs) between isolates from different patients, phylogenetic analysis can accurately reconstruct transmission chains, revealing how resistant strains spread within a sub-region. Integrating this detailed genomic data with epidemiological information creates a powerful tool for public health surveillance, enabling targeted interventions to halt the spread of drug-resistant TB (27). Moreover, to help the various TB control programs in West Africa and governments to get a better perspective of the TB burden in the sub-region, we published a review article that summarizes the burden of TB with an emphasis on DR-TB in West Africa, efforts being made to fight against TB, the challenges facing TB control efforts, and ways to ameliorate these challenges to help win the fight against TB in West Africa (28).

## Conclusion

Tuberculosis remains a major global health issue, particularly in West Africa, which harbors diverse MTBC lineages. Misdiagnosis and drug resistance are major threats to TB control in West Africa, compounded by the probable underestimation of the true prevalence of drug resistance. The TB working group of WANETAM, funded by the EDCTP, is therefore tackling TB in West Africa through equitable and collaborative research among partners, with an emphasis on training, gender balance, and enhancement of laboratory facilities. In West Africa, we focus on (1) improving the diagnosis of childhood TB within national health systems in West Africa by evaluating the sensitivity and specificity of non-sputum based diagnostics that use urine, stool, and tongue swabs, (2) assessing the suitability of new diagnostics by evaluating new diagnostics for West Africa’s unique MTBC diversity, (3) conducting DR-TB surveillance to determine the existing burden of DR-TB in the sub-region to help advise policy, (4) improving laboratory capacity through support for accreditation/certification and supply of essential equipment to regional laboratories, and (5) targeted training or capacity-building for research leadership and technical expertise with an emphasis on gender equality. By bringing Francophone, Anglophone, and Lusophone West Africans together to successfully fight TB and prepare for unexpected future pandemics—through improvement in laboratory infrastructure, training of a critical mass of expertise, and conducting impactful research to advise policy in West Africa—WANETAM has shown that “Yes! We Can End TB” when we work together.

## Data Availability

The original contributions presented in the study are included in the article/Supplementary material, further inquiries can be directed to the corresponding author.

## Footnotes

^1^ https://wanetam.net

^2^ https://aslm.org/resource/whos-slipta-checklist/

^3^ https://thetalentfellowship.org/

## Group members of WANETAM-TB-Network

Presented in Supplementary material.
