# Supplementary material for: Defying barriers to fight tuberculosis in West Africa: a model of equitable partnerships within a research capacity-strengthening network in the subregion
Source: Front Public Health. 2025 Sep 19;13:1590282. doi: 10.3389/fpubh.2025.1590282 (PMC12491211; doi:10.3389/fpubh.2025.1590282)
Supplement: Supplementary file 1 [file Data_Sheet_1.DOCX]

**Title:** Defying barriers to fight tuberculosis in West Africa: A model of equitable partnerships within a research capacity strengthening network in the subregion.

**WANETAM-TB-Network**: Isaac Darko Otchere^1,2,^ Adwoa Asante-Poku^1^, Oluwaseyi Ayoola-Williams^1,3^, Sheila Agyeiwaa Owusu^2,4^, Victory Fabian Edem^2^, Awa Ba Diallo^5^, Adama Sanou^6,7^, Nneka Onyejepu^8^, Fatimata A. Diallo^9^, Antieme C G Togo^9^, Bassirou Diarra^9^, Yaotsè Anoumou Dagnra^10^, Augustine O. Ebonyi^11^, Arnauld Fiogbe^12^,Audrey Forson^13^,Dissou Afolabi^14^, Aderemi Kehinde^15^, Plácido Monteiro Cardoso^16^, Abdoulie Badjan^17,18^, Toyin Togun^2,19,20,+^, Dorothy Yeboah-Manu^1+^, Martin Antonio^2,21,22+^

**Affiliations**:

^1^Noguchi Memorial Institute for Medical Research, University of Ghana, Accra, Ghana

^2^Medical Research Council Unit The Gambia at the London School of Hygiene and Tropical Medicine, Banjul, The Gambia.

^3^Department of Microbiology, University of Ghana Medical School, University of Ghana, Accra, Ghana

^4^Department of Paediatrics and Child Health, School of Medicine, University for Development Studies, Tamale, Ghana

^5^Biological Sciences Department, Faculty of Pharmacy at Cheikh Anta Diop University, Dakar, Senegal

^6^Centre Muraz, Institut National de Santé Publique Bobo-Dioulasso, Burkina Faso

^7^Unité de Formation et de Recherche en Sciences de la Vie et de la Terre, Université Nazi Boni, Bobo-Dioulasso, Burkina Faso

^8^Nigerian Institute of Medical Research, Lagos, Nigeria

^9^University Clinical Research Center, University of Sciences, Techniques and Technologies of Bamako, Bamako, Mali

^10^National Tuberculosis Reference Laboratory, Lomé, Togo.

^11^Department of Paediatrics, Jos University Teaching Hospital and University of Jos, Plateau State, Nigeria.

^12^National Teaching Hospital for Tuberculosis and Respiratory Diseases, Cotonou, Republic of Benin.

^13^Korle Bu Teaching Hospital and University of Ghana Medical School, Accra, Ghana.

^14^National Tuberculosis Program, Republic of Benin.

^15^Department of Medical Microbiology & Parasitology, College of Medicine, University of Ibadan, Ibadan, Nigeria.

^16^Bandim Health Project/National Institute, Bissau Cedex, Guinea Bissau

^17^National Public Health Laboratories, MoH, The Gambia

^18^University of The Gambia Medical School, Banjul, The Gambia

^19^Clinical Research Department, Faculty of Infectious and Tropical Diseases, London School of Hygiene and Tropical Medicine, London, UK

^20^The TB Centre, London School of Hygiene and Tropical Medicine, London, UK

^21^Department of Infection Biology, Faculty of Infectious and Tropical Diseases, London School of Hygiene and Tropical Medicine, London, UK

^22^Centre for Epidemic Preparedness and Response, London School of Hygiene and Tropical Medicine, London, UK
